# Machine Learning for Process Monitoring and Control of Hot-Melt Extrusion: Current State of the Art and Future Directions

**DOI:** 10.3390/pharmaceutics13091432

**Published:** 2021-09-09

**Authors:** Nimra Munir, Michael Nugent, Darren Whitaker, Marion McAfee

**Affiliations:** 1 Centre for Mathematical Modelling and Intelligent Systems for Health and Environment (MISHE), Institute of Technology Sligo, Ash Lane, F91 YW50 Sligo, Co. Sligo, Ireland; 2 Centre for Precision Engineering, Materials and Manufacturing Research (PEM Centre), Institute of Technology Sligo, Ash Lane, F91 YW50 Sligo, Co. Sligo, Ireland; 3 Faculty of Engineering, Athlone Institute of Technology, Dublin Road, N37 HD68 Athlone, Co. Westmeath, Ireland; MNugent@ait.ie; 4 Perceptive Engineering—An Applied Materials Company, Keckwick Lane, Daresbury, Warrington WA4 4AB, Cheshire, UK; Darren_whitaker@amat.com

**Keywords:** hot-melt extrusion (HME), machine learning, drug, polymer, process analytical technology, in/on-line process monitoring, Industry 4.0

## Abstract

In the last few decades, hot-melt extrusion (HME) has emerged as a rapidly growing technology in the pharmaceutical industry, due to its various advantages over other fabrication routes for drug delivery systems. After the introduction of the ‘quality by design’ (QbD) approach by the Food and Drug Administration (FDA), many research studies have focused on implementing process analytical technology (PAT), including near-infrared (NIR), Raman, and UV–Vis, coupled with various machine learning algorithms, to monitor and control the HME process in real time. This review gives a comprehensive overview of the application of machine learning algorithms for HME processes, with a focus on pharmaceutical HME applications. The main current challenges in the application of machine learning algorithms for pharmaceutical processes are discussed, with potential future directions for the industry.

## 1. Introduction

Hot-melt extrusion (HME) is a rapidly growing technology in the pharmaceutical industry, for the preparation of various dosage forms, including granules, pellets, tablets, and implants. The HME process offers many advantages relative to other pharmaceutical processes, one of the major benefits being that HME can enhance the bioavailability and solubility of poorly soluble drugs. Further, as a solvent-free process, it is free of harsh environmental toxicants and no additional step for solvent recovery is required, unlike solvent evaporation and spray drying. HME is also relatively easy to scale-up, and it is a continuous process [1].

As with all pharmaceutical products, polymer–drug extrudates that are produced using HME must undergo rigorous quality analysis and typically undergo thermal, rheological, mechanical, and chemical characterisation. For thermal analysis, DSC and TGA are widely used to measure the percentage of crystallinity, the glass transition temperature (T_g_), and the change in weight. The results of these methods have been used to predict the miscibility, solid state, and stability of the polymer–drug matrix [2,3,4,5,6,7,8,9,10,11,12,13,14,15,16,17,18,19]. Rheological analysis is used to provide information about the behaviour of the polymer–drug system under the high temperature and stresses experienced in the process itself [20,21,22,23]. HPLC is used to monitor the drug/additive content in the extrudate [4,5,14,18,19]. FT-IR, Raman, and NIR have been employed post-production to study the stability and for analysing the drug content in the polymer–drug matrix [2,4,10,18,19,24]. Physicomechanical testing [25,26] and dissolution testing [27,28] are also performed for product quality assurance. The main disadvantage of these off-line, lab-based methods is that there is a long lag between processing and feedback on product quality, which makes process control very challenging. HME is a continuous process, but the long testing time for product quality assurance defeats this advantage of the process.

In 2004, the Food and Drug Administration (FDA) introduced the concept of using process analytical technology (PAT) [29]. The main aim is to improve the understanding of the mechanism of the manufacturing process, enhance process monitoring, and to reduce the processing time. In the literature, spectroscopic techniques, including Raman [2], NIR [30], and UV–Vis spectroscopy [31], have been widely implemented as PAT tools for in/on-line monitoring of the HME process, and an in-line slit die rheometer has also been implemented as a PAT tool in some studies [32,33]. Machine learning (ML) algorithms are generally used to infer the required information from in/on-line collected spectra.

**Figure 1:**
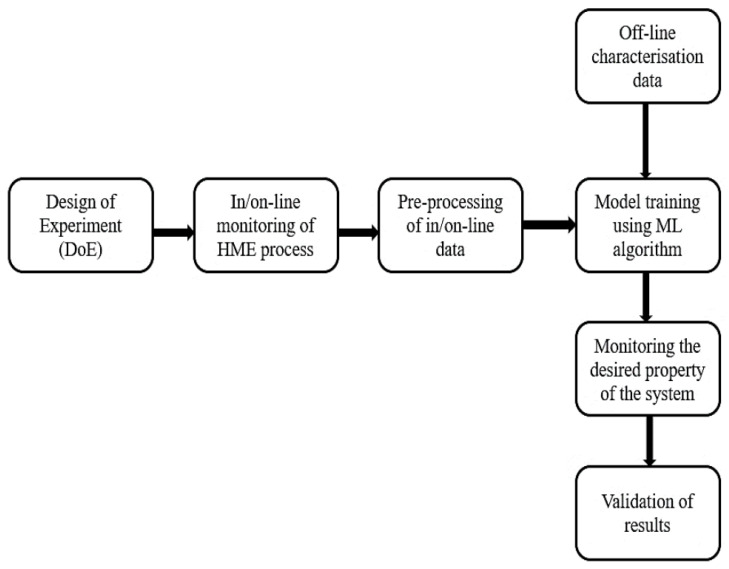
Schematic representation of in/on-line monitoring of HME process with machine learning.

A PAT tool, coupled with a machine learning algorithm, has been established as an effective way to monitor the HME process in real-time. Figure 1 gives a schematic representation of the work flow for in/on-line monitoring of the HME process using PAT tools coupled with machine learning. Since 2004, many research studies have been reported, in which different machine learning algorithms have been applied to in-process data to analyse product and process parameters in real-time. The applications include the monitoring of product critical quality attributes (CQAs), including the following: the solid state of the polymer/drug [2,3]; API/additive concentration [34,35]; degradation of the polymer [36,37]; the particle size of additive/s [31]; and mechanical properties [33]. Other works have examined the monitoring of critical process properties (CPPs), including melt temperature [38], pressure [39], and viscosity [40], and for process fault detection [41].

In recent years, many review papers have been published focusing on different aspects of the HME process [1,15,42,43,44,45,46,47,48,49,50,51,52,53,54,55,56,57,58]. In this review, we focus specifically on the application of machine learning (ML) algorithms in the monitoring and control of the HME process. Greater process sensorization, coupled with algorithms for deriving intelligence from data, are key concepts of the Pharma 4.0 initiative for the digital transformation of the pharmaceutical industry, hence this review aims to establish the current state of the art of the HME process in this respect. We present and discuss the various data analytics and machine learning methods reported for the monitoring and control of HME, including the following: methods for the pre-processing of data, model training/calibration, the ability of the developed model to detect the effect of varying processing conditions, and the performance of models on unseen data. We summarise the contribution that machine learning has made to date in the monitoring and control of the HME process and discuss the main challenges and future potential of the field.

The remainder of the paper is organised as follows: First, a brief introduction is given to machine learning and the main data pre-processing techniques that are relevant to the HME process. The main body of the paper then reviews the applications of (i) PCA, (ii) PLS, and (iii) non-linear machine learning algorithms to the process, followed by the discussion and conclusions.

## 2. Machine Learning

Machine learning (ML) is generally defined as an ability of a computer to learn without being explicitly programmed. Machine learning algorithms train themselves to identify patterns in the data or make predictions based on past data, as opposed to modelling algorithms that are based on the prior physical/chemical knowledge of a system. A machine learning system can be predictive; descriptive (meaning that the system uses the data to explain what happened); or prescriptive (meaning that the system will use the data to make suggestions about what action to take). ML algorithms can be divided into the following three classes: supervised learning, unsupervised learning, and reinforcement learning [59].

### 2.1. Supervised Machine Learning

In supervised learning, algorithms are provided with known/labelled input–output data [59,60]. In other words, supervised machine learning algorithms try to predict the results for an unknown output based on the patterns present in the labelled data set, i.e., the algorithm tries to approximate the mapping function from input to output variables. Regression and classification are categorised under supervised machine learning.

Classification algorithms classify training data into separate categorical classes/groups. All the samples of data in the training set are labelled. The purpose of using classification is to identify the class of future unknown observations. There are the following three types of classification: binary classification with two possible outcomes; multi-class classification with more than two classes; and multi-label classification, whereby each input in the training data is mapped to more than one class [61]. The classification algorithm’s performance is assessed based on how well an algorithm classifies unseen observations into the correct classes. A confusion matrix is created for performance assessment, where the rows represent the true classes and the columns represent the predicted classes. Naïve Bayes, *k*-nearest neighbours (*k*-NN), decision tree, support vector machine (SVM), and random forest (RF) are commonly used classification algorithms [62,63].

In regression, the class of the output variable is continuous numeric. Linear regression methods include methods such as partial least squares regression (PLS), least absolute selection shrinkage operator (LASSO), and ridge regression; while random forest (RF) regression and support vector regression (SVR) are commonly used non-linear regression algorithms. In the literature, the performance of a regression algorithm is generally assessed based on its root mean square error (RMSE), which is based on the difference between the actual and predicted values, and on the coefficient of correlation (R^2^) values.

### 2.2. Unsupervised Machine Learning

Unlike supervised learning, the inputs are not labelled in unsupervised learning and the algorithm is concerned with detecting regularities/patterns in the unlabelled training data [59]. Clustering (e.g., *k*-means and hierarchical clustering) is a well-known class of unsupervised machine learning [60,64]. In clustering, the aim is to find similar subgroups within the data set; all the objects are divided into a certain number of clusters, and inputs with a similar pattern are gathered in the same cluster. Principal component analysis (PCA) is another very common unsupervised machine learning method. It is usually used for dimensionality reduction in data sets with a degree of collinearity between the input variables [65]. In PCA, the input variables are transformed into a new set of input features, which are linear combinations of the original variables. These new features or ‘principal components’ (PCs) successively explain the variance in the input data, such that most of the variation in the data can be captured by a small number of PCs and redundant input features, representing noise in the data set, can be ignored.

### 2.3. Reinforcement Learning

In reinforcement learning (RL), the learning process is different from supervised and unsupervised learning. Reinforcement learning is an agent-based learning process, whereby a ‘reward’ is associated with each learning action by the agent. An RL process proceeds with trial and error, and an agent learns through its interaction with the environment. To achieve the given task, i.e., to maximise the reward signal, it takes different actions, and experiences many failures and successes [66,67].

## 3. Pre-Processing Techniques for In-Process Spectral Data

Raw spectral data, collected using spectroscopic methods, typically undergoes pre-processing before applying a chemometric model [68]. During the process, spectral data can be affected by nuisance factors, including physical interruptions and faulty apparatus. These factors can reduce the signal-to-noise ratio and resolution [69]. Other undesirable features of raw spectra are baseline shifts and a complex background. Baseline shifts are caused by the scattering of the light, resulting from the interaction of spectra with the sample particles [68]. Undesired scatter effects can dominate the desired information (e.g., chemical information) in the spectra [70]. These undesired spectral variations can increase the complexity and reduce the accuracy of the model [69]. The main goal of pre-processing techniques is to remove the unwanted features from the spectra.

For in-process spectral data, the following two groups of pre-processing techniques dominate the literature: scatter correction and spectral derivatives. Multiplicative scatter correction (MSC), extended MSC (EMSC), extended inverse MSC, de-trending, normalisation, and standard normal variate (SNV) belong to the scatter-correction group; these methods are used to correct baseline shifts and trends in the baseline. The spectral derivative group includes Norris-Williams (NW) and Savitzky-Golay (SG) and are used for smoothing and for reducing the noise effects [68,71]. The most common pre-processing techniques used in the literature are MSC, SNV, derivatives, and SG.

MSC is used to remove undesired scatter effects. MSC defines a reference spectrum, which is commonly the average spectrum of the calibration set [72]. MSC is a two-step process involving the estimation of shifting and scaling correction coefficients [68]. After MSC, all the spectra have the same offset and amplitude [68,70]. SNV is also used to eliminate baseline shifts. SNV and MSC are quite similar to each other, but in SNV, a spectrum is mean-centered and then scaled by its standard deviation [68]. Smoothing is also a pre-processing method used to increase the signal-to-noise ratio. The moving average, where each spectral point is substituted by the average of *m* neighbouring points, is the simplest smoothing method (*m* is defined as the width of the smoothing window) [71]. Savitzky-Golay (SG) is a popular smoothing method that performs local least squares regression on the spectral data [72,73]. Differentiation is usually applied after applying smoothing methods. Derivatives are used to increase the spectral resolution and to eliminate the background effects. The first derivative eliminates constant baseline shifts, while the second derivative eliminates linear shifts in the spectrum, along with eliminating constant baseline shifts [72,73,74]. For more in-depth reading on these methods to understand the differences and similarities, the reader is directed to the review article [68] and these papers [69,71,75].

## 4. Application of PCA for In-Process Monitoring of Critical Quality Attributes (CQAs)

PCA is a technique for dimensionality reduction, which falls under unsupervised machine learning [65,76,77]. Details on how PCA works with PAT tools for monitoring pharmaceutical processes (other than HME) can be found in [78,79,80], and the detail of the algorithm is not repeated here. PCA has mostly been utilised in the HME literature to monitor the effect of varying processing conditions on the solid state of the drug. The drug solid state significantly influences the dissolution rate and bioavailability of the drug, with an amorphous form of the drug exhibiting a higher dissolution rate than the crystalline form.

**Figure 2:**
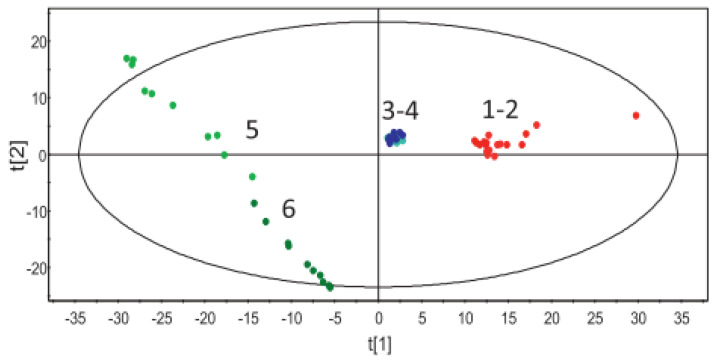
Scores scatterplot for in-line Raman spectra recorded at different temperatures. (Reproduced with permission from Almeida et al., International journal of pharmaceutics; published by Elsevier, 2013) [70].

Almeida et al. [70] used PCA to monitor the effect of screw speed and barrel temperature on the solid state of metoprolol tartrate (MPT) extruded with ethylene-vinyl acetate (EVA). Six different batches were processed under different combinations of temperature (90, 110 and 140 °C) and screw speed (90 and 110 rpm). All Raman spectra were pre-processed using SNV before developing a PCA model. Three separate clusters were identified along the first principal component (t[1], see Figure 2), representing the Raman spectra from the extrusion batches processed at three different temperatures. The clustering is caused by the reduction in the drug crystallinity due to an increase in temperature. The PCA results indicated that the effect of screw speed on the solid state of MPT was not very prominent at the lower processing temperatures (90–110 °C), as there was no clear separation of the points relating to different screw speeds in these temperature clusters (see Figure 2, spectra from experiments 1–2 and 3–4). However, when MPT was processed above its melting point (140 °C), an increase in screw speed was found to have a significant effect on the solid state of MPT. At 140 °C, MPT was present entirely in melt form—thus increasing the screw speed produced a more significant temperature difference in the product, which resulted in separation of the high and low screw speeds in the PCA scores scatterplot (see Figure 2, spectra from experiments 5–6). These results were confirmed by off-line DSC results. Similar results were obtained with PCA analysis applied to in-line NIR spectra at the same processing conditions.

Saerens et al. [10] used PCA to monitor the effect of different MPT concentrations (10% and 40%), extrusion temperatures (100, 120, and 140 °C), and screw speeds (80 and 160 rpm) on the solid state of MPT along the barrel. For this purpose, in-line Raman spectra were collected from the following different sections of the extruder: the feeding section (S1) and five-barrel sections (S2, S3, S4, S5, and S6). SNV was used as a pre-processing step. For 10% MPT, a PCA scores scatterplot indicated no difference in the solid state of MPT with varying barrel temperatures, as the points relating to all the spectra were clustered together. This is because a solid solution was formed for all the temperatures. For 40% MPT at 100 °C and 140 °C, three separate clusters were identified along PC2; these clusters grouped the spectra collected from different barrel sections. This clustering was due to the difference in the drug crystallinity at different barrel sections. For 10% MPT at 120 °C, doubling the screw speed did not affect the final solid state of the extrudate. On the contrary, for 40% MPT at 140 °C, increasing the screw speed from 80 to 160 rpm significantly affected the solid state of the drug, as indicated by the PCA results; at higher screw speeds, a solid solution was formed.

Saerens et al. [2] used PCA to monitor the effect of drug loadings (30%, 40% and 50%), processing temperature (130 to 150 °C), and screw configurations on the solid state of celecoxib (CEL) extruded with Eudragit^®^E PO. DSC and XRD were used for off-line characterisation. First, they ran PCA on off-line data collected from DSC and XRD. A PCA scores scatterplot demonstrated the following two separate clusters: the first cluster grouped all the extrusion experiments where CEL was present in crystalline form, and the second cluster grouped all the extrusion experiments where CEL was present in amorphous form. The in-line Raman spectra were pre-processed using SNV. After PCA, all the spectra grouped into two clusters. However, unlike in the case of XRD and DSC, the Raman spectra from the extrusion experiments with 30% CEL (extruded at 130 °C) could not be classified into any of the groups, indicating the presence of partial crystalline CEL at these conditions. The predictive ability of a PCA classification model was assessed on an independent validation set. For this purpose, the in-line Raman spectra were classified into two groups, crystalline or amorphous, according to the off-line DSC and XRD results. Separate PCA models were developed for both groups. For the validation set, the class membership of new observations was decided by using a Coomans’ plot [81]. A Coomans’ plot for the validation set correctly identified CEL in the product as either crystalline or amorphous using the in-line Raman spectra. Further, under conditions where CEL was semi-crystalline in the extrudate, the Raman spectra could not be classified as either crystalline or amorphous. The same procedure was repeated with off-line XRD and DSC. For the XRD data, three data points from 50% CEL extruded at 130 °C, and for DSC, three data points from experiments with 50% CEL and one from the experiment with 40% CEL, could not be classified in any of the classes as CEL was present in semicrystalline form in these samples. PCA analysis indicated that the effect of screw configuration on the solid state of the drug was insignificant. This study showed the better sensitivity of Raman spectroscopy coupled with PCA over the conventional off-line methods DSC and XRD, to precisely monitor the solid state.

**Figure 3:**
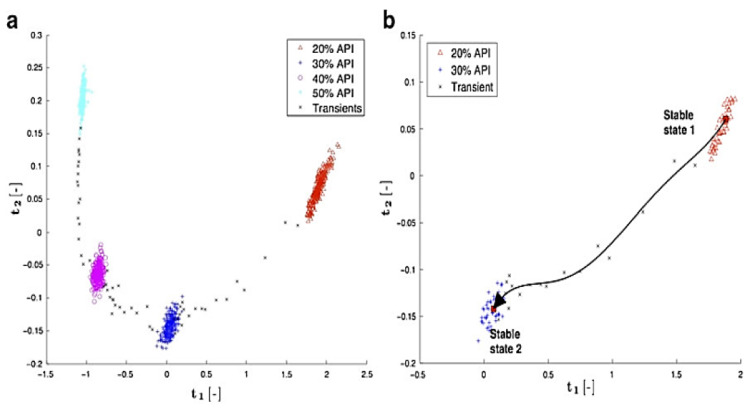
(**a**) Different API concentration with feed rate, (**b**) real-time monitoring to evaluate the time required for the transition from stable state 1 to stable state 2. (Reproduced with permission from Markl, D et al., AAPSPharmSciTech; published by Springer Nature, 2013) [82].

Markl et al. [82] used PCA to investigate the effect of varying feed rates on paracetamol concentration in a paracetamol and calcium stearate (CaSt) mixture in real-time, using in-line NIR in the die section. SIPAT (a commercially available software solution by Siemens AG, Munich, Germany) was used to align the spectra with process measurements of barrel temperature and pressure, and screw speed. SIMCA-Q was used to analyse the data, and the processed data was visualised in SIPAT. Without any pre-processing, raw NIR spectra were used for developing the PCA model. On a PCA scores scatterplot, four separate clusters were observed, relating to the presence of different API contents. The plot showed that at different feed rates, different API contents were present in the die section. They further highlighted that on varying the feed rate, the API contents varied in the process stream, which affected the PC1 score. Thus, from the scores scatterplot, the time to reach the steady-state can be determined, i.e., by comparing the score value of PC1 and reference feed rate in real-time (see Figure 3).

**Figure 4:**
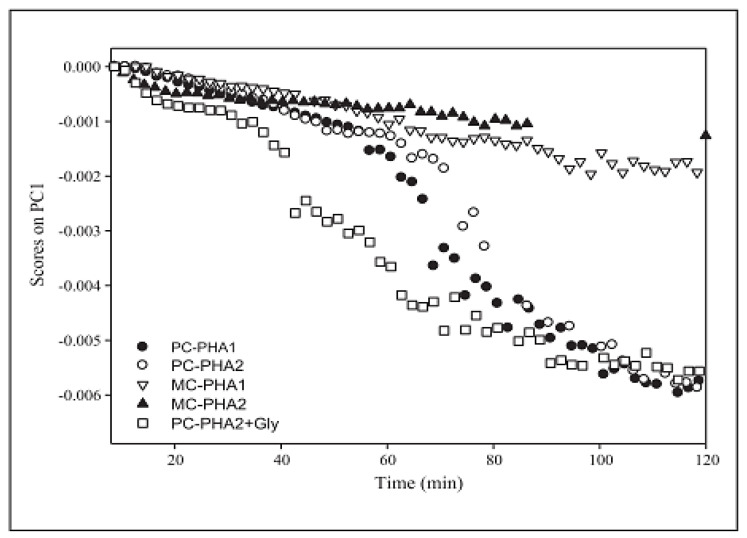
PC1 scores plot with time. (Reproduced with permission from Montano Herrera et al., new biotechnology; published by Elsevier, 2013) [37].

Other works have also shown the significance of monitoring the PC1 score in real-time to monitor various aspects of the HME process. Chirkot et al. [30] investigated the effect of varying processing conditions on the uniformity of the extrudate in processing 5% ibuprofen with 95% Kollidon at 160 °C. They varied the screw speed (200, 400, and 600 rpm) and feed rate (1.5 and 2 kgh^−1^). A PC scores scatterplot showed higher variability in the spectra at higher screw speeds and very small variability in the spectra with changing feed rate. Montano-Herrera et al. [37] used PC scores to monitor the degradation of four different polyhydroxyalkanoates (PHAs) (mixed and pure cultures) during extrusion. NIR spectra were pre-processed using MSC, second derivative, and mean centring. The PC1 scores were plotted against time and a change in the gradient of the plot against time was used as an indicator of degradation. A scores plot revealed higher spectral changes with time for pure cultures than for mixed cultures, which, in other words, suggested higher degradation for pure cultures than mixed cultures (see Figure 4).

In summary, PCA has been shown to be a useful technique for monitoring drug solid state in real-time with in-line spectroscopy data, provided that previous off-line characterisation has been carried out to identify the solid state associated with different clusters in the data. The method allows for rapid identification of the effect of process parameters on the solid state, which is valuable for process control purposes. Further, monitoring of the PC1 score in real-time has been shown to be useful to identify when a process has reached steady state, to monitor the effect of processing conditions on variability in the extrudate, and to monitor in-process degradation as well.

## 5. Application of PLS for In-Process Monitoring of Critical Quality Attributes (CQAs)

PLS regression is a multivariate linear regression method that is suitable for highly collinear data. Analogous to PCA, it involves a linear transformation of the data set, allowing for dimensionality reduction to a reduced number of ‘latent variables’ (LV), which are linear combinations of the original variables. General details on the workings of the PLS algorithm applied to PAT data for pharmaceutical process monitoring can be found in [78,79,80,83]. In pharmaceutical processes, PLS is primarily used to predict the concentration of the drug, although it has also been used to predict polymer blend contents, degradation of the polymer, the particle size of fillers in the polymer matrix, and mechanical properties of the polymer extrudate in non-pharma HME processes.

### 5.1. In-Process Monitoring of the Drug Content

Dadou et al. [84] used PLS to measure the concentration of two model drugs, ramipril (RMP) and hydrochlorothiazide (HCTZ), in fixed dose combinations, using an in-line Raman spectrometer. Five different concentrations of HCTZ and RMP were extruded with Eudragit^®^E. The Raman spectra from these concentration levels were used to develop a PLS calibration model. All the Raman spectra were pre-processed using SNV and first derivative, followed by SG and MSC, before using them for developing the PLS model. The actual amount of drug in all the extruded samples was measured using off-line HPLC. The PLS model was developed using pre-processed Raman spectra regressed against the off-line measured drug content. The predictive ability of the PLS calibration model was assessed using an independent validation set. The concentration levels of HCTZ and RMP used in the validation experiments were different from the ones included in the calibration model. The PLS model showed a good ability to predict drug content in real-time, with an RMSEP (RMSE of prediction, i.e., on unseen validation data) of 1.237% and 1.007% for HCTZ and RMP, respectively.

Tumuluri et al. [85] used PLS to predict the concentration of clotrimazole and ketoprofen, used as model drugs in different concentrations with polyethylene oxide (PEO). The first set of experiments was conducted using a pilot-scale extruder, while the second set of experiments (used for model validation) was conducted using a lab-scale extruder. Different concentration levels were used for the second set of experiments. Six Raman spectra were recorded for each concentration level, for both ketoprofen and clotrimazole. All the Raman spectra were pre-processed using the second derivative. Separate PLS calibration models were developed for ketoprofen and clotrimazole, using on-line collected spectra. It should be noted that calibration models were developed by regressing on-line collected spectra versus the theoretical drug concentration, and the actual drug content in the extrudate was not evaluated. The performance of the PLS models was assessed by using the RMSEP achieved on the validation set. The RMSEP values achieved for ketoprofen and clotrimazole were 0.94% and 0.97%, respectively. The results of this study indicate good transferability of the PLS model to a different extrusion machine with different drug loadings.

Saerens et al. [9] applied in-line Raman spectra coupled with PLS to predict the concentration of MPT extruded with Eudragit^®^ RL PO. The MPT concentration was varied as 10%, 20%, 30% and 40%. The spectra were pre-processed using mean centring, SNV, and Savitzky-Golay. A total of forty spectra were collected from each extrusion run; twenty spectra from each concentration level were used to train the PLS model; and a remaining twenty spectra from each extrusion run were used as the validation data set. An RMSEP value of 0.59% was achieved with two latent variables on the validation set.

In another study, Saerens et al. [11] used Kollidon^®^SR as a polymer carrier and monitored the concentration of MPT in real-time using in-line NIR coupled with PLS. Three different extrusion runs with MPT 20%, 30%, 40% (*w*/*w*) were performed. The pre-processing steps were multiplicative scatter correction (MSC) followed by the second derivative. A total of sixty spectra were collected and used to train the PLS model. The predictive ability of the PLS calibration model was examined more rigorously here, using an independent validation set. For the validation set, separate extrusion runs were performed at different times, using the same MPT concentrations. The RMSEP of the PLS model for the validation set was 1.54% with R^2^ = 0.97.

Vo et al. [34] used FT-NIR coupled with a PLS model to monitor the in-line concentration of ketoprofen in Eudragit. Seven different concentration levels of ketoprofen (from 40% to 60%) were used. A total of eighty-five spectra were collected from these seven concentration levels, and all the spectra were pre-processed using Norris second derivative and SNV methods. Seventy-three spectra were used to train the PLS model, and the remaining twelve spectra were used as a validation data set to analyse the predictive performance of the PLS calibration model. The PLS model with five factors achieved an RMSEP value of 0.62% for the validation set. To further investigate the robustness of the calibrated PLS model, extrusion trials were carried out at 120 °C and the feed rate was maintained at 100 g/h. However, they induced ±10 °C variations in the temperature to simulate the temperature variations during the actual process. The PLS model proved robust enough to accurately predict the API concentration when the temperature varied between 110 and 130 °C.

Chirkot et al. [30] used in-line NIR coupled with a PLS model to monitor the concentration of ibuprofen used as a model drug, with Kollidon used as a polymer carrier matrix. Firstly, ibuprofen (2.5–10%) was used at a fixed feed rate of 1 kgh^−1^, and at a screw speed of 200 rpm. NIR spectra were pre-processed using second-order derivatives. The PLS calibration model showed a good correlation (R^2^ of 0.992), with an acceptable error of 0.4%.

Kelly et al. [86] used in-line NIR coupled with a PLS model to monitor the concentration of carbamazepine (CBZ), used as a model drug, and polyethylene glycol (PEG) as a plasticiser extruded with Kollidon^®^ VA 64 as a polymer carrier. The purpose of this research work was to monitor the concentration of CBZ and PEG in real-time. The concentration of CBZ and PEG was varied from 5.0 to 27.5 and 5 to 20 *w*/*w*%, respectively. The second derivative was used as a pre-processing step. Separate PLS models were trained using sixty-six in-line collected spectra to monitor the CBZ and PEG contents. The predictive performance of the PLS calibration model was tested on an independent validation set that included twelve spectra from the concentration levels of CBZ and PEG, which were not included in the PLS calibration model. For CBZ, a PLS model with four latent variables showed excellent performance and achieved an RMSEP of 0.672%. For PEG, a PLS model with six latent variables achieved an RMSEP of 1.06%. A higher number of latent variables were required for PEG than for CBZ, as the PEG peaks were less prominent in the spectra as compared to CBZ. This work indicates the ability of the PLS model to predict the drug and plasticiser content simultaneously. However, these experiments were carried out at very low screw speeds and low throughput, resulting in a long residence time. In the actual production process, the residence time is much shorter than in this study.

Overall, these studies indicate a good ability of the PLS model coupled with PAT to monitor drug content accurately. The PLS models also showed good predictive ability under varying processing conditions. However, the processing conditions that were used for validation were not very different from those used for model calibration.

### 5.2. In-Process Monitoring of Cocrystal Concentration

Kelly et al. [87] used in-line NIR coupled with a PLS model to study the effect of temperature (80 and 90 °C), screw speed (20, 30 and 40 rpm), and screw configuration on the cocrystal formation of ibuprofen and nicotinamide. All the spectra were pre-processed using second derivatives. PXRD (powder X-ray diffraction) was used off-line to determine the relative cocrystal conversion of ibuprofen. For a PLS model, with the data sets from the different screw configurations, a good correlation (0.903) between the actual and predicted cocrystal purity was found. However, this PLS model could not generalise well for varying screw speed. The author suggested that the poor generalisation performance was due to temperature effects, as temperature influences absorption in the NIR region. To investigate this, they calibrated a second PLS model using only the spectra collected at 90 °C, and two screw configurations (with medium and high mixing intensity). This model performed better than the previous one, as indicated by the better correlation coefficient (0.999).

Wood et al. [88] used in-line NIR spectroscopy coupled with PLS to study the co-crystal concentration in a mixture of cocrystal and pure API. In this study, two different APIs, ibuprofen (IBU) and carbamazepine (CBZ), were used with nicotinamide (NIC), which was used as a co-former. A PLS regression model was developed using the in-line NIR spectra of a mixture of 1:1 IBU/CBZ-NIC cocrystal and pure ibuprofen/carbamazepine. The PLS calibration model was developed using twenty samples, while the validation set consisted of ten samples. They used nine different NIR spectral regions along with using five different types of pre-processing treatments, including first and second derivatives, Savitzky-Golay smoothing, Norris smoothing, and SNV. The PLS model for IBU-NIC, which included the spectral range 7450–7000 cm^−1^, and used SNV, second derivative, and NS as pre-processing steps, achieved the best results. In the case of CBZ-NIC, a PLS model with 9000–8500 cm^−1^ and using SNV, second derivative, and SGS as pre-processing steps performed better than other PLS models. The PLS model with two latent variables for IBU-NIC achieved better predictive accuracy than the PLS model with two latent variables for CBZ-NIC.

Similarly, Karimi-Jafari et al. [89] also monitored the cocrystal concentration of ibuprofen and nicotinamide by using in-line Raman in combination with a PLS model. They also calibrated the PLS models using different spectral ranges and pre-processing steps. Finally, a PLS model, with five latent variables incorporating a full spectral range and using SNV as a pre-processing step, achieved the lowest RMSE value of 0.834% for the validation set.

The works [88,89] highlight that the use of different spectral regions and different pre-processing steps results in differences in the predictive accuracy. A better predictive performance can be achieved by selecting the most relevant (according to the target variable) spectral region. To date, the selection of optimal pre-processing steps has been presented as a trial-and-error procedure, specific to the particular system being investigated.

### 5.3. In-Process Monitoring of the Polymer Blend Concentration and Filler Content

Rohe et al. [90] used in-line NIR coupled with PLS to monitor the concentration of PE/PP, where the PE content varied from 0 to 100%. Twenty spectra were collected from each composition and pre-processed using different combinations of the following steps: averaging a number of spectra, smoothing, data reduction (reduction in spectral resolution by averaging neighbouring wavelength values to a single point), differentiation, and MSC. The leverage correction method was used to test the performance of the PLS model. Using leverage correction validation, all the samples are included in the calibration set. A PLS model, with five latent variables, and using spectral averaging and smoothing as pre-processing steps, yielded the lowest RMSEP value of 0.38% among all the models. Moreover, the transferability of the PLS calibration model was also investigated. For this purpose, thirteen different PLS models were trained: eleven PLS models included measurements conducted at different times, and two models only included measurements conducted at the same time. These latter models showed better performance. This study highlights the difficulty of transferability of the PLS model from one set of measurements to a new set.

Barnes et al. [91] employed FT-NIR for monitoring the concentration of magnesium hydroxide, used as filler in LDPE. The concentration of Mg(OH)_2_ was varied from 0 to 15 wt.%, at screw speed 15 rpm and processing temperature 200 °C. Twenty spectra from each concentration level were used to develop a PLS calibration model, while five spectra from each concentration level were used as a validation set. The PLS model with two latent variables achieved a standard error of prediction (SEP) of 0.27 wt.% in the validation set. In the same study, they extruded seven random EVA copolymers with varying VA monomer contents (2 to 43 wt.%) and used Raman and FT-NIR to monitor the EVA copolymer contents. Baseline correction was applied to the FT-NIR spectra, while for the Raman spectra, MSC was used as the pre-processing step. For FT-NIR, a PLS model with three latent variables, and for Raman spectra, a PLS model with five latent variables, achieved an SEP of ±0.38 wt.% and ±0.187%, respectively, for the validation set.

### 5.4. In-Process Monitoring of Polymer Degradation

Montano-Herrera et al. [37] extended their work on PCA to monitor the degradation of four different polyhydroxyalkanoates (PHAs) (see Figure 4), by using PLS regression to predict the presence of C–H groups in the degradation products. The NIR spectra were pre-processed using MSC, second derivative, and mean centring. They used the spectral range 4300–6200 cm^−1^ (identified via the PCA analysis) to build a PLS model. A model with five latent variables achieved an RMSECV of 0.0126, and the quantification of C–H groups predicted by the PLS model correlated with off-line proton nuclear magnetic resonance spectroscopy (HNMR).

Guo et al. [36] used PLS with in-process Raman to monitor the degree of degradation of polypropylene (PP). PP was repeatedly extruded fifteen times to investigate the ability to monitor the increasing degradation of PP in real-time. The off-line GPC results indicated a decrease in the molecular weight with successive extrusion runs. Raman spectra in the range of 1600–600 cm^−1^, after baseline corrections, were used to calibrate a PLS model. The PLS model to predict the degree of degradation was calibrated using in-line Raman spectra and off-line GPC data. The PLS calibration model included data from the 1st, 4th, 7th, 10th and 13th extrusion, and data from other extrusion runs were used in the validation set. A PLS model with four latent variables showed good accuracy in predicting the degree of PP degradation and achieved an RMSEP value of 1.7228%.

### 5.5. In-Process Monitoring of the Mechanical Properties of Polymer Product

Witschnigg et al. [92] used in-line FT-NIR coupled with a PLS model to predict the Young’s modulus of PP filled with 5% organo-clay. During extrusion, two different screw geometries (differing in number and position of kneading elements), termed as geometry 1 and geometry 2, and screw speeds of 100 to 300 rpm were used. The NIR spectra were pre-treated using mean centring and SNV. The performance of the PLS model was evaluated using RMSECV and R^2^ values. For geometry 2, more significant deviations in the actual and predicted Young’s modulus values were observed. Furthermore, the authors optimised a PLS model with mean-centring and normalisation, to predict the interlayer distance, i.e., the spacing between the nano-clay particles. A good correlation was achieved for both screw geometries. Finally, on-line drawing force was also measured and a PLS model was optimised with mean-centring and MSC to predict this response variable. Table 1 lists the R^2^ and RMSECV values for the PLS model to predict Young’s modulus, interlayer distance, and drawing force.

### 5.6. In-Process Monitoring of Filler Particle Size

Whitaker et al. [31] used PLS and linear discriminant analysis (LDA) (a classification algorithm) with UV–Vis spectroscopy to monitor the particle size (D50) of β-TCP (beta-tricalcium phosphate (Ca_3_ (PO_4_)_2_) extruded in a packaging grade PLA. Two different particle sizes of β-TCP (5 and 30 µm) were used and processed separately. To check the robustness of the models, the experiments were repeated on different days. The experiments performed on the first day were used to train the models for LDA and PLS; the second- and third-day extrusion runs were used as the validation set. The predictive accuracy of PLS was reasonable; for 30 µm particles, the predictions were 31.4 ± 0.64 and 30.40 ± 0.2, and for 5 µm particles, the predictions were 13.096 + 0.16 and 7.62 + 0.51, for second- and third-day extrusion runs, respectively. Furthermore, to analyse the ability of PLS and LDA models to detect when the concentration of larger particles exceeds a set upper limit (as a model for detecting a high concentration of agglomerated particles), different-sized particles were mixed in different ratios. The ratio of 5:30 µm was varied as 19:1, 18:2, and 15:5% *w*/*w*, while keeping the total concentration of the additive at 20% *w*/*w*. The LDA model showed excellent ability and classified all the spectra correctly, except two. Similarly, the PLS model also performed very well and demonstrated a maximum deviation of 0.02% from the expected values. They further studied the transferability of the PLS and LDA calibration models by using an independent validation set, which consisted of a data set obtained from the extrusion of β-TCP with a medical-grade PLA. The LDA model was relatively accurate in classifying the spectra into classes relating to either large or small particles in the system. Similarly, the PLS model showed good accuracy for 30 µm particles but yielded higher variations for 5 µm particles. This work showed that models for additive particle size could be trained using a cheap grade of PLA and applied to processing of the more expensive medical-grade PLA.

These research works on the monitoring of polymer blend concentration, polymer degradation, mechanical properties, and particle size may be useful for further investigation in pharmaceutical HME processes where a blend of polymers is desired as the carrier matrix; thermally sensitive polymers are used; the agglomeration or size of additive particles is a concern; or in the manufacture of implant forms where mechanical properties are important.

## 6. Application of PCA and PLS for Process Fault Detection and Statistical Process Control

Statistical process control (SPC) enables quick insights into process data through the use of graphical presentation of the data [93]. SPC charts are generally used to monitor the critical process variables, to see if they are within the defined limits. In the case of PCA-based methods of SPC, two important metrics are SPE (standard prediction error) and T^2^ thresholds [94]. An SPE value higher than the threshold value is interpreted as a breakdown in the data, which is an indication of a potential fault in the system. Similarly, a T^2^ value higher than the threshold value is an indication that the process is away from being ‘normal’ [41]. Besides, D^2^ [95] is also used for detecting outliers after applying PCA.

Liu et al. [96] used PCA for the early detection of variations in the melt viscosity as an indication of fault during the extrusion of low-density polyethylene (LDPE). An in-line slit die rheometer was used to record the in-line shear viscosity. Due to the non-linear nature of the polymer extrusion process, a non-linear PCA algorithm based on serial principal curves and radial basis function (RBF) networks was used. Fault-free data (i.e., from a process with consistent viscosity) were used to build a non-linear PCA model, and the performance of the model to detect the faults in the extrusion process was examined using data from a process where viscosity variations were deliberately induced. The performance of the non-linear PCA model was compared with linear PCA and ICA-PCA (independent components analysis PCA). The performance of the models was assessed based on both T^2^ and D^2^ statistics. The results showed that the ICA-PCA model performed better than the linear PCA model; however, the non-linear PCA model outperformed both the methods and detected the change/disturbance in the process much earlier.

Kazmer et al. [97] examined PLS and PCA models to detect faults in a tubing extrusion process. They used nine different manipulated variables, including different blend ratios of the polymers (LDPE and LLDPE), screw speeds, barrel temperatures, die temperatures, linear-pull speeds, tubing internal pressures, temperatures and flow rates of the water bath, and extrudate free lengths between the water bath and die lip. They trained four different PCA and PLS (PCA1, PCA2, PLS1, PLS2) models, where each model had a different set of input variables (selected from machine sensors, micrometer data, and process variables) and differed from each other in terms of complexity. Separate validation experiments were performed, in which eighteen different types of disturbances were introduced, and the previously trained PCA and PLS models were used to detect the faults in the extrusion process. All the models showed reasonably good performance in detecting the faults related to viscosity changes, pressure, screw speed, temperature changes, etc. However, the models were not very efficient in detecting physical variations that did not affect the extrusion process dynamics, e.g., slight deviations in the extrudate diameters were not readily detected by the models. The results showed that PCA outperformed PLS in detecting the variations in the system. This work also indicated that the performance of the models could be increased by adding more input variables in training the models, as all the models showed better sensitivity in detecting the changes related to the variables used for training the models.

**Figure 5:**
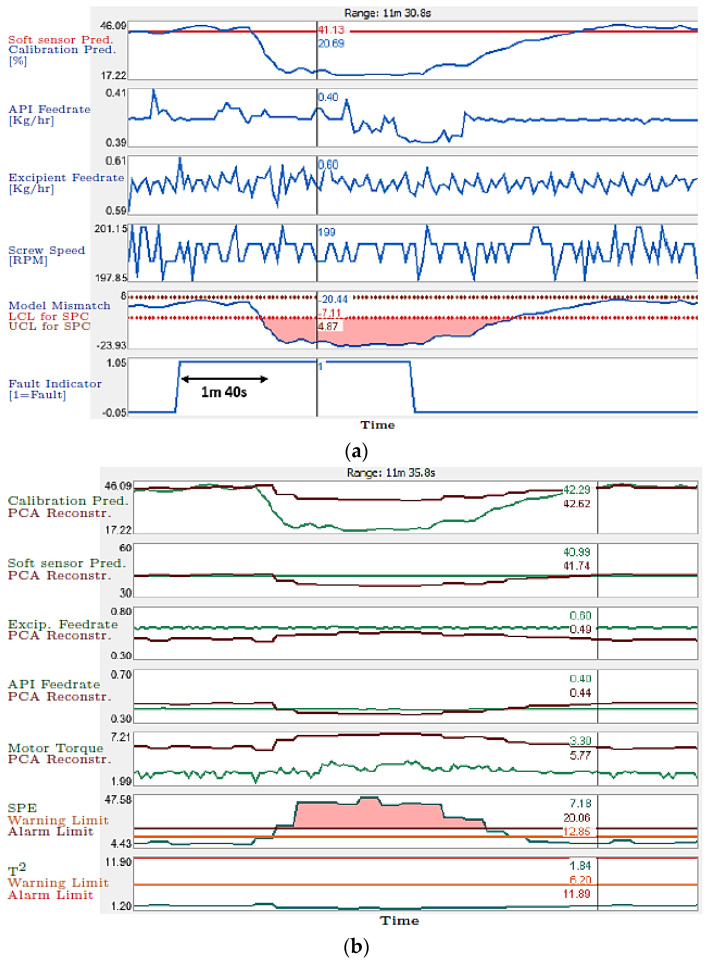
(**a**) SPC data, (**b**) PCA data SPE and T2. (Reproduced with permission from Tahir et al., computer and chemical engineering; published by Elsevier, 2019) [41].

Tahir et al. [41] developed two different soft sensors for the prediction of the concentration of paracetamol used as a model drug, with Affinisol used as a polymer carrier matrix. The first PLS model was based on in-line Raman spectra, and the second model, called a hybrid soft sensor model, was built using feeder process data, to predict the dynamic concentration of the API at the end of the extruder outlet. Both the models showed good accuracy in predicting API concentration. The predictions of these models, along with the process data, were used by the PCA model with 2 PCs and an SPC model. These two models were used as tools for the detection of various faults in the process. To compute the ‘model mismatch’ signal, a Shewhart control chart [98] was developed. This chart computed the upper and lower control limits (UCL, LCL) of the process. Different disturbances were induced in the process, including the following: (1) API powder accumulation in the barrel zone; (2) material deposition on the Raman probe; and (3) the presence of an impurity in the API. For the case of API powder accumulation, the PLS model correctly predicted the reduced API percentage in the extrudate. The hybrid soft sensor model was unable to detect this change however, as this model was based on the amount of API and excipient in the feeders. The SPC detected a mismatch in the signal, caused by divergent model predictions in under 2 min. PCA was also able to detect the fault as observed by the higher SPE value (see Figure 5). Similarly, in all other cases, the SPC and PCA models were able to detect the process disturbances.

## 7. Application of Non-Linear ML Algorithms for HME Process

### 7.1. Non-Linear ML Algorithms to Monitor CPP

Non-linear machine learning algorithms have been applied in monitoring critical process parameters, such as melt temperature, melt pressure, and melt viscosity, for the HME process. Melt temperature determines the process thermal stability and melt quality, and influences the production rate [99,100]. Abeykoon et al. [101] developed a non-linear static model using the fast recursive algorithm (FRA) to model the temperature profile across the melt. Finally, an FRA model with 12 terms and of the 6th order, which achieved the lowest RMSE of 2.89 for the validation set, was selected. Further, optimisation algorithms were applied to the model to optimise the process settings. The results showed a reduction in thermal variations when optimised process conditions were used compared to the pre-set experimental conditions used earlier. In this study, screw speed and melting zone temperature were identified by the FRA model as the most influential parameters to affect the melt temperature. In another study, Abeykoon et al. [38] used FRA coupled with a backward elimination method to develop a non-linear dynamic model to predict the die melt temperature. They tried different models, and a second order model with 20 terms achieved the best predictive accuracy. The metering zone temperature was identified as the most important barrel zone temperature to affect the melt homogeneity and temperature level.

Melt pressure is another important process parameter to monitor during the HME process. Abeykoon et al. [39] used FRA coupled with backward elimination and developed a non-linear model to predict the static pressure and a linear model to predict the dynamic pressure using different processing conditions. Both models showed good results; the screw speed and barrel zone temperatures were identified as the most influential parameters to affect the melt pressure.

Melt viscosity is also considered an important process parameter to monitor the quality and homogeneity of the product. McAfee et al. [102,103] and Liu et al. [40], in a series of studies, developed soft sensor models with a ‘predictor-corrector’ structure to monitor the melt viscosity, with different materials and using different equipment. These soft sensors used information on the process settings to estimate the resulting melt viscosity using grey-box modelling approaches. This estimated viscosity is used to predict the process melt pressure, and the error between the predicted and measured melt pressure is fed back to continuously correct the viscosity estimates, despite changes in the feed material, etc. The proposed soft sensors achieved good predictive ability on different grades of material than those used in model training. Kugler et al. [104] developed a soft sensor model using an artificial neural network (ANN) to predict the small changes in viscosity caused by variations between batches of material in production. This work showed good performance of the soft sensor model to predict the viscosity changes in real-time. However, they observed significant deviation between the actual and predicted values at some points, due to fluctuations in torque and pressure signals.

Good predictions of critical, but difficult-to-measure process parameters, such as thermal homogeneity and melt viscosity, have been achieved with non-linear algorithms. Such models also yield insight into the most important process variables and facilitate optimisation of process settings. Some works have shown that such models can sometimes be robust to changes in material batches and grades, depending on the design. However, such models are also more complex, may require large data sets to train, and may be more likely degrade in performance over time.

### 7.2. Application of Non-Linear ML Algorithms for On/In-Line Monitoring of Product Quality

A small number of research works have been reported that show the potential of alternative methods (other than PCA and PLS), such as random forest (RF), ANN, *k*-NN, and SVM in HME, to predict product drug content, dissolution profile, and mechanical and dimensional properties.

Regev et al. [105] used an ANN model coupled with an evolutionary algorithm to investigate the effect of barrel temperature, screw speed, and feed rate on the dissolution profile, puncture strength, and drug content for vaginal film. Dapivirine (DPV), used as a model drug, was blended with PEG, HPC, and vitamin acetate to manufacture vaginal films using the HME process. Eighty percent of the experimental data was used to train the ANN model, while the predictive performance of the model was investigated on the unused 20% of the data. Different structures were explored and, finally, a fully connected, feed forward network, with a single hidden layer, was found to be the best performing model. The final ANN model achieved percentage errors of 15.46%, 5.32% and 8.71% for drug content, puncture strength, and dissolution profile, respectively, for the 20% unseen data. A surface response analysis of the ANN model indicated that changes in the barrel temperature significantly affected all three response variables; the screw speed affected the puncture strength more significantly than the drug content and dissolution; while changes in the feed rate were found not to significantly affect any of the three targeted response variables. Furthermore, they used an evolutionary algorithm to optimise the process parameters. The percent difference between the predicted and experimental data was less than 1% for dissolution, drug content, and puncture strength, after using the optimised barrel temperature, screw speed, and feed rate values suggested by the evolutionary algorithm.

Mulrennan et al. [33] used PCA coupled with random forest regression to predict the yield stress of PLA processed at a range of different temperatures, screw speeds, and feed rates. During the process, pressure and temperature data were captured, including pressure drop along an instrumented slit die, which was used to estimate the shear viscosity during processing. For the final calibration model, only the pressure data and the shear viscosity estimates were used as very little variation was captured in the melt temperature data. Eighty percent of the data was used to train the model, and the remaining 20% of the data was used as unseen data, to test the predictive performance. Four different models were trained, including PCA–random forest, PCA–bagging, random forest, and bagging, to predict the yield stress of PLA. PCA–random forest showed the best performance, as indicated by the lowest RMSE value; however, all the other methods also performed reasonably well.

Garcia et al. [106] used different regression models, including *k*-NN, SVR, and MLR, to predict the inner diameter (ID) and outer diameter (OD) of the tube during a tubing extrusion process. In this study, data was captured on fifteen process variables, including four barrel temperatures, four die temperatures, the hopper temperature, cooling tank temperatures, screw speed, vacuum pressure, and pulling force. They predicted the internal (ID) and outer (OD) diameter by using simple *k*-NN, two distance-weighted *k*-NN models (termed as *k*-NNRw1 and *k*-NNRw2), a linear regression (LR) model, and three different SVR algorithms (linear kernel (SVR-1), polynomial kernel (SVR-2), and a radial basis function kernel (SVR-RBF)). To predict the OD, *k*-NNRw1 and SVR-RBF achieved the lowest RMSE values. To predict the ID, *k*-NN and SVR-RBF yielded the lowest RMSE values. However, to predict the ID, all the methods based on *k*-NN performed reasonably well.

Zhu et al. [107] used both in-line Raman and NIR in a data fusion technique to monitor PP/PS blend concentration. Data fusion is defined as a method to combine data from different sources having the following three levels: low-level, combining all the raw data; mid-level, combining only features extracted from the raw data; and high-level fusion, where only the results from individual models are combined. Zhu et al. used low- and mid-level data fusion methods. In this study, they used three different PP grades and two different PS grades. Pre-processing methods for the NIR and Raman spectra included baseline correction, and minimum and maximum normalisation. One calibration set and three different validation sets were used. For the calibration set, the concentration of PP was changed from 95% to 5 wt.%, while for the validation sets, the concentration of PP was changed from 90 to 10 wt.%. For validation set 1, the same grades of PP and PS were used as for the calibration set, while for validation set 2 and set 3, the PP and PS grades used were different than those used in the calibration set. They compared the performance of PLS, ANN, and extreme learning machine (ELM) regression models using NIR and Raman data separately, and then using the low- and mid-level data fusion techniques. Table 2 summarises the results for all the models for the validation set. In all the approaches, the linear PLS produced poorer predictive accuracy than the non-linear ANN and ELM methods. Mid-level fusion produced better results, as it yielded lower RMSEP values for both ANN and ELM than for low-level fusion. Nevertheless, low-level fusion also performed reasonably well for ANN and ELM.

Table 3 and Table 4 summarise the application of machine learning algorithms for pharmaceutical and polymer HME processes, respectively.

Although not heavily investigated in the HME process to date, non-linear ML techniques have been shown to be useful in the monitoring of complex product quality attributes using heterogenous process data.

## 8. Discussion

The application of machine learning algorithms clearly has an important role in monitoring product and process parameters that are relevant to achieving a robust pharmaceutical HME process. In particular, the methods of PCA and PLS enable the rapid identification of critical quality attributes such as solid state and quantification of drug contents, as well as having a role in detecting faults in the process. In this section, we evaluate the main challenges in developing and applying ML to quality control in industrial pharmaceutical processes, and also consider the future directions in ML developments for pharmaceutical HME under Industry 4.0.

### 8.1. Improvement of Conventional Linear Methods

For the HME of a polymer–drug matrix, it is evident from the literature that PCA and PLS have been used in almost all applications, not least because of their tried and tested effectiveness for chemometric applications and suitability for application with relatively small data sets—as is usually the case in pharmaceutical process development. The majority of research works address the interpretation solely of spectroscopic data (typically NIR or Raman spectra) to monitor chemical properties, usually at fixed process conditions. Although PCA and PLS have shown promising results in various applications, these methods have some limitations. PCA and PLS may not perform well with non-linear data [110]. Also, the use of PLS and PCA may, in some cases, reduce the access or the interpretability of the data, as information on which regions of the spectrum are responsible for the majority of the variation is obscured.

Recently, a number of researchers have examined PLS extensions to improve performance in non-linear settings and eliminate redundant features, resulting in a sparser and more interpretable model, and often with improved accuracy. It has been shown that feature selection methods coupled with multivariate regression methods, such as PLS, can significantly improve the predictive performance over a simple PLS model [111]. The genetic algorithm with PLS (GA-PLS) has been used to detect relevant spectral regions and eliminate redundant regions. However, one of the limitations associated with GA-PLS is that when the number of wavelength features are high (usually greater than 200), the detection of relevant spectral regions becomes difficult. To overcome this challenge, backward interval (bi-PLS) following GA for feature selection can be used. Bi-PLS splits the spectrum into a given number of intervals and performs backward elimination. With the removal of one interval at each stage, the performance of the model is improved (i.e., RMSECV reduces). Ultimately, the bi-PLS method selects the most-relevant spectral region [112]. Marini et al. [113] used bi-PLS coupled with a GA to predict the enantiomeric excess in both mandelic acid and ketoprofen in pellets. The performance of a PLS model that was developed using the features selected by bi-PLS coupled with GA was compared with full PLS (including all the features and a full spectrum range). For both ketoprofen and mandelic acid, the PLS model with fewer features achieved better predictive accuracy than the full PLS model. Table 5 summarises the results of this study.

Shah et al. [111] proposed using a statistical pattern analysis (SPA) feature-based soft sensor for the analysis of in-process spectral data. In SPA, instead of monitoring the process variables, the process operation status is monitored by monitoring various statistics of key process variables [114,115]. In this study, firstly, the whole spectrum was divided into various non-overlapping segments using synergy interval segment PLS (SiPLS). Secondly, they extracted different summary statistics from each spectrum segment, and used these to train a PLS model. The hyperparameters used to tune the PLS model performance included using different numbers of segment intervals, different numbers of PCs, and different summary features from the wavelength segments. Using this methodology, they analysed the NIR spectra of the pharmaceutical tablet data set, and compared this method with the SiPLS, full PLS model, and LASSO. The SPA feature-based model showed better performance than the full PLS model and LASSO. The benefit of using the SPA feature-based soft sensor is that it utilises information from the entire spectrum but reduces the number of variables for training the model.

### 8.2. The Role of Sensor Integrity and Location

Models based on machine learning algorithms are trained using process inputs that are recorded using physical sensors, such as pressure and temperature sensors, and in/on-line spectrometers. The performance of the machine learning models depends heavily on the accuracy of these physical sensors. In the case of using an in-line spectrometer, it is important to make sure that the changes in the spectral features represent the changes materials go through during the process. The spectral changes should be sufficient enough to monitor the desired system property, and the signal strength should be high enough to distinguish between the instrument noise and the spectral changes [116]. Similarly, wrongly positioned or poorly calibrated physical sensors used during the process can affect the predictive performance of the machine learning models. Verstraeten et al. [117] developed a PLS model to monitor API concentration during a bottle-filling step of a pharmaceutical process. However, when they plotted the predicted NIR assay as a function of time, continuous fluctuations were observed in the assays, which was in contradiction with ultra-performance liquid chromatography (UPLC) results. Further investigation was made by using a CFD model, which confirmed the presence of a recirculation zone very close to the location of the NIR probe—indicating that the flow was not in fully developed conditions when the NIR measurements were taken. To address this issue, they increased the distance between the NIR probe and interface inlet. Assay predictions as a function of time for optimised arrangement were then consistent and did not exceed the assay control limits of 95% and 105%.

### 8.3. Potential for Non-Linear ML Methods

It has been shown that ML algorithms have a role not only in the analysis of spectroscopic data, but also in monitoring the process health more generally, e.g., in identification of fluctuations in feeding, melt temperature, and viscosity, etc. The application of more-complex ML algorithms in other pharmaceutical processes indicates the future potential of ML in HME in a more holistic way to develop process models for the purposes of process optimisation and control. A few brief examples are given here to indicate the potential of more-sophisticated ML algorithms for process modelling and monitoring.

For other pharmaceutical processes, ANNs, in particular, have shown good predictive ability in different applications, including predicting granule size distribution (GSD) for a dry granulation process [118], particle size for a wet granulation process [119], and the prediction of tensile strength for a tabletting process [120].

Dengler et al. [121] used machine learning models for the quality control of a medical product formation system, to identify different types of errors and to minimise the false rejection rate. They used different machine learning algorithms at different stages of the process, including anomaly detection, decision tree, support vector machine and a convolutional neural network. Overall, the proposed approach was able to detect the defective components and reduce the false rejection rate to an acceptable limit.

He et al. [122] used both linear and non-linear machine learning algorithms, including the following: DNN, SVM, RF, MLR, PLS, *k*-NN, and light gradient boosting machine (LightGBM), to predict the particle size and polydispersity index of nanocrystals. In both cases, out of all the models, LightGBM yielded the lowest MAE (Mean Absolute Error).

One of the main challenges associated with the application of ML algorithms for the pharmaceutical HME process is related to the ‘small data’ problem, especially at the development stage. Most of the more complex, non-linear machine learning algorithms require large training data for model development; however, some recent works indicate potential with small data sets. Harms et al. [123] used an extended iterative optimisation technology (EIOT) approach to analyse the API content in a small data set during a continuous drug manufacturing production (CDMP) process. EIOT is an optimisation technique based on the Lambert-Beer’s law for spectral decomposition. This method typically includes pure component spectra collected before analysis and mixture spectra collected during analysis [124]. They first compared the performance of NIR and Raman to monitor low drug concentration, and PCA analysis indicated a better ability of Raman to detect the low API content at varying process conditions than NIR. The Raman spectra with 1% API concentration were further optimised using extended iterative optimisation technology. A good agreement was observed between API concentration predicted by EIOT, and off-line API concentration found by HPLC. This method can be used as an alternative to an ML method at the early development stage, when the API supply is not enough to perform regression via PLS, ANN, etc., as EIOT requires very limited training data.

Yang et al. [125] compared the performance of the following six machine learning algorithms: MLR, PLSR, RF, *k*-NN, and SVM, with a deep neural network (DNN) for prediction of the drug release profile from data on the formulation of different oral sustained-release matrix tablets (SRMT), and oral fast-disintegrating films (OFDF). Specifically, the study aimed to predict the disintegration time for OFDF, and the cumulative dissolution profiles for SRMT. In both cases, DNN achieved better accuracy than conventional machine learning methods. SVM and *k*-NN showed reasonable performance in the prediction of the release profile of OFDF. However, none of the conventional machine learning methods could demonstrate reasonable accuracy for SRMT. This study demonstrates the possibility of using DNN to predict pharmaceutical formulations using a small data set.

Blazhko et al. [70] proposed a method for data augmentation (i.e., extending a data set where it is not sufficiently large for training an ML model), based on augmenting IR spectra with physical distortions. It was found that the method can replace pre-processing when combined with DNNs for classification and is especially successful for small data sets. The concept of data augmentation may be a useful avenue for further research in the application of DNNs to HME problems and may also be useful to solve the problem of which pre-processing steps to apply to give the best performance.

A barrier to the adoption of non-linear and complex ML algorithms is the issue of regulatory acceptance. To this end, further research will be needed to ensure the robustness of such models, and the issue of ‘explainability’—understanding how and why the algorithm works—is likely to be a key factor in future adoption by the industry.

### 8.4. Transferability Challenges for ML Models

To implement machine learning methods on a production scale, ideally, one model should be robust enough to be used under different conditions, as the calibration and validation of a model for different conditions is time and cost consuming. As described above, a few studies have been reported in which the transferability of machine learning models was studied in HME processes. Whitaker et al. [31] studied the transferability of PLS and LDA calibration models for monitoring filler particle size in different grades of PLA (packaging and medical grade). They achieved good model transferability, attributed to the similar optical properties of the melt in each case. McAfee et al. [102,103] and Liu et al. [40] investigated the transferability of the soft sensor models with a ‘predictor-corrector’ structure (using feedback from pressure sensors), to monitor melt viscosity when applied to different materials and different equipment and found good transferability of the soft sensor models. However, for monitoring physio-chemical properties, such as API content, there is no obvious relationship between such properties and a real-time process measurement, such as melt pressure.

Rohe et al. [90], in their study to monitor polymer additive contents using HME, investigated the transferability of a PLS model that was trained on NIR data collected at one time to data that was collected at different times, and could not get good accuracy. However, Tumuluri et al. [85] investigated the transferability of a PLS-based model to predict the concentration of API in different extrusion equipment. A PLS model was calibrated using on-line Raman data from a pilot-scale extruder, and the transferability of the PLS model was studied on a lab-scale extruder. A number of the parameters were adjusted in the calibration model to compensate for differences in the extruders, such as, the thickness of the extruded film, and differences in the path length because of film undulation. After these adjustments, the PLS model achieved good predictions for the lab-scale machine.

However, the transferability of machine learning models for the pharmaceutical HME process has not been addressed in most of the studies. The performance of the machine learning models has primarily been assessed on a validation set where the processing conditions used for the validation set were not significantly different from the ones used for the calibration model. In the field of chemometrics more generally, significant research is ongoing to avoid the need for rebuilding a model from scratch for different conditions. One of the approaches that has been investigated in the chemometrics literature is to use adaptively updating calibration models. In the adaptive methods, the existing model requires the tuning of hyperparameters in order to adapt to new data. Recursive partial least squares regression (RPLS) is one such adaptive method. In RPLS, after acquiring new data, the previously calibrated model is updated by adjusting the hyperparameter/s, which controls the level of down-weighting of old training data. However, the implementation of adaptive methods is complex and requires extensive effort to tune the updating of the adaptive hyperparameters of the model. Another method that can be used to update the calibration model for new data is to use a ‘moving window’ modelling approach. Adaptive methods require extensive training data to calibrate a model, while the moving window modelling approach is fast, can be used with small historical data, and is easy to implement [126].

### 8.5. Validation of ML Models

In the literature, most of the authors have used the same approach for the validation of the model, i.e., using the RMSE, RMSECV, and RMSEP values to assess the predictive ability of the model. These metrics are a good way to assess the quantitative performance of the models but are not sufficient for regulatory compliance of the validation of analytical procedures under ICH Q2 [127]. According to these guidelines, the validation methods should include evaluation of trueness, precision (repetitive and intermediate), accuracy, linearity, specificity, and robustness of the model.

Based on these requirements, The Société Francaise des Sciences et Techniques Pharmaceutiques (SFSTP) introduced the concept of an “accuracy profile” for the validation of analytical procedures. An accuracy profile-based validation approach is ICH Q2 compliant but is more demanding than the model evaluation metrics commonly reported in the literature. The approach is based on ‘total error’ (which is the sum of bias and standard deviation) and introduces the concept of a tolerance interval (β-tolerance interval). The accuracy profile is a reliable tool to evaluate the model’s actual performance and assess what kind of results the same analytical procedure will produce when used in the future for routine analysis [108,128,129]. A detailed summary of this approach has been explained in [128,130,131]. Further, a detailed review of different validation methods specifically for the application of NIR in pharmaceutical processes has been published [129]. Here, we present the few research works that have been reported to date in the literature specific to the application of accuracy profile validation methods in HME.

Saerens et al. [108] evaluated the validation performance of different PLS models developed using Raman spectra, to predict the concentration of MPT in an HME process. During the process, the MPT concentration was varied and also the extrusion trials were conducted over three days with two different operators. Four separate PLS models were developed using different spectral pre-processing steps and using all or an average of the spectra. In this case, MCR pre-processing and an average of 10 consecutive spectra showed a better accuracy profile than other PLS models, as it was the only model where β-expectation tolerance intervals remained within the defined acceptable limit (±10). The authors further investigated the robustness of the best performing PLS model by introducing minor changes in the processing conditions. The resulting model performance was evaluated by using the Q^2^ statistic and by using two F-tests (regression model significance test and the lack-of-fit test). The results of these tests validated that the model is robust to the small changes in the process conditions.

Netchacovitch et al. [109] used an accuracy profile for the validation of models developed from in-line Raman spectra, to predict the concentration of itraconazole (ITZ) in Soluplus. The amount of API used as a reference to calibrate the PLS model was determined in the following two ways: (1) by using an off-line confocal Raman microscope, and (2) by determining the theoretical value of the API based on its amount in the sample. The purpose of this step was to investigate the effect of the reference method on the model performance. Training and validation experiments were performed at different times. A linear univariate model and multivariate PLS models were developed to quantify the ITZ concentration. In the case when the API concentration was measured using a confocal Raman microscope, the multivariate PLS model showed a better accuracy profile (acceptance limit ±15) result than the univariate model. The accuracy profile results showed that 95% of the future measurement would fall within the defined limits using a multivariate PLS model. For both the models, when the models were developed using the theoretical API amount the accuracy decreased. These results highlight the importance of using absolute concentrations instead of the theoretical values of the samples.

Almeida et al. [35] monitored and optimised the concentration of piroxicam (PRX) in Kollidon^®^ VA 64 using in-line UV–Vis coupled with PLS. They developed one calibration model and two validation data sets; all three data sets (calibration and validation sets) included data from extrusion runs performed on different days. Normalisation was performed as a part of the pre-processing step, and a PLS model with four latent variables was selected as a final model. Validation was based on the accuracy profile strategy and ICH Q2 (R1) validation criteria. The parameters used for performance assessment of the models included the following: trueness, linearity, precision, limit of quantification and range, total error, and uncertainty. The accuracy profile for both the validation sets showed that with an acceptance limit of ±5, 95% of the future measurement would fall within the defined limits.

## 9. Conclusions

The application of machine learning in pharmaceutical processing is a rapidly developing field, with many potential benefits for process optimisation and control. A well-designed machine learning model can speed up the development process, aid optimisation of the process, reduce the process cost, enhance product consistency, reduce process faults, and enable rapid validation of product quality. However, the use of machine learning algorithms for pharmaceutical HME is relatively new and is as yet underdeveloped. Most of the works reported in the literature have been conducted to predict/monitor the solid state of the polymer–drug extrudate and the API concentration. Few works have been published to date on predicting the final properties of the product such as, degradation of the polymer–drug matrix, mechanical properties and rate of loss of mechanical properties, drug release profile, etc., from in-process data. Recent works examining the application of more-complex ML models, both in HME and more widely in pharmaceutical processing, indicate that with careful design of the sensing system, the experimental procedures, and the modelling algorithms that prediction of such properties from in-process data may be possible in the future. Further, the application of machine learning for automating process control, for example, by using reinforcement learning, has not yet been explored in the literature. Future work should be in the direction of examining the suitability of different machine learning methods, their robustness, and limitations to predict and control the final properties of the polymer–drug matrix. It is stressed that if such models are to meet the industrial requirements for product validation that appropriately rigorous model validation procedures should be applied.

## Figures and Tables

**Table 1:** RMSECV for Young’s modulus, interlayer distance and drawing force using PLS.

	Young’s Modulus		Interlayer Distance		Drawing Force	
	R^2^	RMSECV	R^2^	RMSECV	R^2^	RMSECV
Geometry 1	97.70%	30 MPa	93.44%	0.0.13 nm	94.59%	2.64 mN
Geometry 2	90.55%	94 MPa	93.51%	0.019 nm	97.50%	1.98 mN

**Table 2:** Summary of result for all validation sets.

	Validation Set	ELM	ANN	PLS
Data		RMSEP	RMSEP	RMSEP
Raman spectra	Validation set 1	0.978	1.691	10.536
	Validation set 2	2.357	3.13	25.755
	Validation set 3	1.313	2.232	13.916
NIR spectra	Validation set 1	1.024	1.221	2.372
	Validation set 2	2.186	2.381	3.079
	Validation set 3	3.124	3.507	2.5311
Low-level data fusion (sample = 500, spectra = 657, NIR spectra = 125, Raman = 532)	Validation set 1	0.658	1.087	1.601
	Validation set 2	0.95	1.291	2.02
	Validation set 3	1.74	1.838	5.119
Mid-level data fusion (sample = 500, spectra = 10, 5 features each from NIR and Raman)	Validation set 1	0.992	0.941	1.915
	Validation set 2	1.411	1.375	2.459
	Validation set 3	1.68	1.617	5.45

**Table 3:** Application of ML for pharmaceutical HME process.

Algorithm Used	In/On-Line Monitoring	Purpose	Pre-Processing	RMSE on Unseen Data	Polymer	Drug	Software Used	Reference
PCA	Raman and NIR	Solid state	SNV	-	EVA	MPT	SIMCA P+	[70]
	Raman	Solid state	SNV	-	Eudragit	MPT	SIMCA P+	[10]
	Raman	Solid state	SNV and mean centring	-	Eudragit	CEL	SIMCA P+	[2]
	Raman	Solid state	SNV	-	Eudragit	MPT	SIMCA P+	[3]
	Raman	Fault detection	-	-	Affinsole	Paracetamol	PharmaMV (Perceptive APC)	[41]
	-	API concentration	-	-	Calcium stearate	Paracetamol	SIMCA-Q	[82]
PLS	Raman	API concentration	Second derivative	ketoprofen = 0.94%, clotrimazole = 0.97%	PEO	Ketoprofen, Clotrimazole	Grams™	[85]
	Raman	API concentration	SNV, SG	0.59%	Eudragit	MPT	SIMCA P+	[9]
	NIR	API concentration	MSC, second derivative	1.54%	Kollidon	MPT	SIMCA P+	[11]
	NIR	Co-crystal concentration	Second derivative	R^2^ = 0.99	Nicotinamide	Ibuprofen	TQ Analyst™	[87]
	NIR	Co-crystal concentration	SNV, Second derivative, NS and SGS	0.95% (Ibuprofen), 3.53% (Carbamazepine)	Nicotinamide	Ibuprofen and Carbamazepine	TQ Analyst™	[88]
	Raman	Co-crystal concentration	SNV	0.83%	Nicotinamide	Ibuprofen	MATLAB	[89]
	Raman	API concentration	MCR	1.09%	Eudragit	MPT	SIMCA P+	[108]
	UV-Vis	API concentration	Normalisation		Kollidon	Piroxicam	MATLAB	[35]
PLS	FT-NIR	API concentration	Norris second derivative, SNV,	0.62%	Eudragit	Ketoprofen	TQ Analyst™	[34]
	Raman	API concentration	-	-	Soluplus	Itraconazole	MATLAB	[109]
	NIR	API/plasticiser concentration	Second derivative	PEG = 0.67%, CBZ = 1.06%	Kollidon	Carbamazepine	TQ Analyst™	[86]
	NIR Raman	API concentration API concentration	Second derivative SNV, ID, MSC, SG	0.40% RMP = 1.007% HCTZ = 1.237%	Kollidon Eudragit	Ibuprofen RMP HCTZ	TQ Analyst™ SIMCA	[30] [84]
ANN	-	Dissolution profile, puncture strength and drug content	-	8.71, 15.46 and 5.32	PEG, HPC, and vitamin E	Dapivirine	Python	[105]

**Table 4:** Application of machine learning for polymer HME process.

Algorithm Used	In/On-Line Monitoring	Purpose	Pre-Processing	RMSE on Unseen Data	Polymer	Reference
PCA	Slit die	Fault detection	-	-	LDPE	[96]
	-	Fault detection	-	-	LDPE, LLDPE	[97]
PLS	NIR	Additive concentration	Spectral averaging and smoothing	0.38%	PP/PE	[90]
PLS	FT-NIR	Filler concentration	-	0.27%	LDPE	[91]
PLS	FT-NIR, Raman	VA monomer contents	Baseline correction (for FT-NIR spectra), MSC (for Raman spectra)	0.38% (FT-NIR), 0.187% (for Raman)	EVA	[91]
PLS	Raman	Degradation	Baseline correction	1.72%	PP	[36]
PLS	NIR	Degradation	MSC, second derivative, mean centring	0.0126	PHA	[37]
LDA and PLS	UV-Vis	Particle size	-	-	PLA	[37]
PLS	NIR	Mechanical properties	Mean centring, SNV, normalisation	-	PP	[92]
PCA-Random forest	Slit die	Yield stress	-	0.25%	PLA	[33]
*k*-NN, SVR, LR	-	Inner and outer diameter of tube	Normalisation	0.00965 (outer diameter), 0.00107 (inner diameter)	-	[106]
ANN, PLS, and ELM	NIR and Raman	Polymer blend concentration	Baseline correction, and normalisation	0.6583 (best result)	PP/PS	[107]

**Table 5:** Summary of bi-PLS-GA and final PLS model result.

Drug	Original Features	Features Selected by Bi-PLS	Features Selected by GA	RMSEP of Final PLS Model	RMSEP of PLS Model (with All Features)
Ketoprofen	1661	196	9	2.12	2.32
Mandelic acid	1391	121	31	4.57	6.87
